# Supplementary material for: Combining formal methods and Bayesian approach for inferring discrete-state stochastic models from steady-state data
Source: PLoS One. 2023 Nov 13;18(11):e0291151. doi: 10.1371/journal.pone.0291151 (PMC10642793; doi:10.1371/journal.pone.0291151)
Supplement: Attachment — Submitted filename: Review Comments for PONE-D-23-14562.pdf [file pone.0291151.s001.pdf]

Editorial suggestions for "*Combining formal methods and Bayesian approach for inferring discrete-state stochastic models from steady-state data*

Line 76: Change "idea" to "ideas"

Line 101: Phrasing: "further elaborated in towards shedding light", I have no idea what this means.

Line 201: Replace "on" with "of"

Line 202: "Central Limit Theorem it guarantees to achieve desired statistical accuracy". Please rephrase. I'm not sure what this means.

General comment: Is it possible to move the figures closer to where they are discussed in the text?

Line 292: Replace "long enough experiments" with "experiments of sufficient length"

Line 304: "We" should not be capitalized

Line 335: Replace "Different to this" with "On the other hand"

Line 377: Insert: "the" before "one-"

Line 379: Replace "point" with "points"

Line 382: Insert "the" before both "Sequential" and "Approximate"

Line 390: Not sure what "with regarding their weights" means

Line 405: Insert "a" after "use"

Line 441: Replace "wrt." with "with respect to". This occurs multiple times in the paper. Everyone knows what you mean, but you should probably avoid such an abbreviation in a formal journal article

Line 444: Replace "index" by "indices"

Page 13: I would suggest that you insert a blank line above "Fig 4". Without this blank line, the "c)" gets lost in the text. It took me a few minutes to figure out which part of your figure was c).

Line 490: Should "Nested" be capitalized?

Lines 504 and 507: Other "wrt" occurrences

Page 15: See note for Page 13 above.

Page 16: Table 1 should probably be tabbed to the right. Also , insert "with" before "6" in the first line of the caption

Page 528: Insert "the" before "two"

Line 556: Another "wrt"

Line 560: "corollary of that scaling up" should be rephrased

Line 565: "Lemma" shouldn't be capitalized

Page 17, caption for Fig 6: Insert “a” before each color

Page 18: Should Proof 1 be italicized?

Line 589: Replace “distribution” with “distributions”

Line 590: “Lemma” shouldn’t be capitalized

Line 604: The word “lossy” is used. I’ve never heard of this word.

Line 608: Insert “its” before “own”

Lines 614 – 615: Should “p,q” read “[p,q]”? Should “OK, Failed” read “[OK, Failed]”?

Line 618: Insert “one” after “allows”

Caption for Fig 7: Again, insert “a” before all colors

Line 637: I’m not sure what “over-approximates well” means

Line 648: “ares” should read “areas”

Line 648: Start a new paragraph with “See”

Proof 2: Should this be italicized? Replace “equals” with “is equal to”

Lines 669 – 686: Check the indenting on the second line of each subpoint

Line 690: Insert “one” after “allows”

Line 707: Replace “is” with “in”

Line 714: Replace “to” with “from”

I hope this helps. Congratulations on a well-written article.
