# Supplementary material for: Combining formal methods and Bayesian approach for inferring discrete-state stochastic models from steady-state data
Source: PLoS One. 2023 Nov 13;18(11):e0291151. doi: 10.1371/journal.pone.0291151 (PMC10642793; doi:10.1371/journal.pone.0291151)
Supplement: Attachment — Submitted filename: response_letter.pdf [file pone.0291151.s002.pdf]

Response to Reviewers

Subject: **Submission of a revised manuscript for evaluation**

Dear Editors,

please find enclosed the revision of the manuscript previously titled *Combining formal methods and Bayesian approach for inferring discrete-state stochastic models from steady-state data*, by Julia Klein, Huy Phung, Matej Hajnal, David Šafránek, and Tatjana Petrov, submitted as a research article to the *PLOS ONE* journal for consideration of publication. All co-authors have seen and agree with the revised manuscript's contents.

First of all, we would like to thank the editors for giving us the opportunity to revise the manuscript. We also thank all reviewers for valuable comments that have significantly helped us to improve the paper. All changes in the manuscript (and the supplementary text) are emphasised in blue colour and described in responses to reviewers comments attached to this letter.

We would like to thank the editors and the reviewers in advance for looking into the revised material.

Yours faithfully,

Tatjana Petrov and David Šafránek on behalf of all the authors.
August 2, 2023

Corresponding authors:

Tatjana Petrov
Department of Computer and Information Sciences
University of Konstanz, 78464 Konstanz, Germany
e-mail: tatjana.petrov@uni-konstanz.de, phone number: +49-7531-88-4565

David Šafránek
Faculty of Informatics
Masaryk University, Botanická 68a, 60200 Brno, Czech Republic
e-mail: safranek@fi.muni.cz, phone number: +420549494476

Review Response

We would like to thank to both the reviewers for valuable comments. Below we give our reactions on the individual comments and describe the relevant changes in the manuscript. The original reviewers comments are typeset in italic.

Reviewer #1

The article is well-written and worthy of publication. I see no mathematical errors. The manuscript contains numerous grammatical, phrasing and spelling errors that need to be corrected prior to publication.

We thank the Reviewer 1 for their positive assessment of our work. We proofread the newly submitted version for grammatical, phrasing and spelling errors.

Reviewer #2

The authors present a framework for estimating the parameters of a discrete time Markov chain given only the label of the final state. The concepts are demonstrated through four simulation and case studies. The content is presented in a very rigorous framework and highly technical language.

Given that PLOS One targets general audience, I believe the manuscript needs a significant attempt to clarify the writing. I have the following specific suggestions. I suggest not using acronyms in the abstract (i.e., BSCC). Additionally, the concept of formal method needs to be defined or given an appropriate reference (line 60).

We have attempted to clarify the writing throughout the text. Specifically, we spelled out the acronyms in the abstract. Moreover, in the abstract and elsewhere applicable, we added terms that may be more accessible to a reader who is not working in the narrow field of formal methods. For instance, we used the term 'terminal components' instead of 'bottom strongly connected components', before introducing the technical term (in the abstract and introduction). We added a clarification and reference for the concept of formal methods (lines 91-95).

From my understanding, the estimation also relies on knowing the structure of the DTMC in order to write down all of the polynomials of the parameters. However, I did not understand from the current manuscript how this assumption comes into play. Please clarify the assumptions of the framework in the appropriate section. Additionally, how can a practitioner hypothesise the connections in order to use the estimation methods?

We thank the reviewer for this important remark. We address this important point at three different places in the manuscript:

- Already in the abstract, we rephrase so to make it more clear why the assumptions of the framework are relevant in practice.
- The submitted version of the paper started by outlining the general technical problem of parameter inference for discrete-time Markov chains. In the revised version, we first start by motivating the problem in more general terms that are closer to the practitioner's points of view, to real-world situations where the connections can be hypothesised (lines 1-19) in the newly submitted version with marked changes)
- In the introduction (lines 20-37) in the newly submitted version with marked changes), we spend a paragraph illustrating more concretely how our method is practically useful when modelling biological systems.
- When introducing the case study of social feedback in honeybee colonies (subtitle 'Social feedback in honeybee colonies'), we reiterate how and why our method is especially relevant for modelling biological systems. Then, we illustrate our methodology over a real-world case study of honeybee collective defence.

- Line 17: suggestion – don't repeat the phrase after the acronym is introduced

Done.

- Suggest rephrasing lines 19-35 for clarity

We rewrote parts of the introduction so to improve clarity.

- Lines 36-47: references? These are strong claims

We added references on parameter identification and the performance of MCMC.

- Line 84: What's SMT?

We clarified what SMT means by writing the term out and adding a footnote with a short explanation.

- Line 101: typo? "elaborated in towards"

Done.

- Line 390: typo "with regarding their weights."

Done.

- Line 453: missing word " would allow to explore"

Done.

Additional suggestions sent to us in pdf form, without specifying which Reviewer

Editorial suggestions for "Combining formal methods and Bayesian approach for inferring discrete-state stochastic models from steady-state data

Line 76: Change "idea" to "ideas"

Done.

Line 101: Phrasing: "further elaborated in towards shedding light", I have no idea what this means.

Done. We hope that by correcting the grammar of the sentence, it is now clear to the reader that we introduced the mentioned methodology in an earlier paper and then elaborated on it in a subsequent paper with the aim of explaining the defence mechanism of honey bees.

Line 201: Replace "on" with "of"

Done.

Line 202: "Central Limit Theorem it guarantees to achieve desired statistical accuracy". Please rephrase.

Done. We rephrased and extended the sentence to emphasise that the Central Limit Theorem ensures that the sample mean is a reliable estimate for the population mean, such that the desired level of accuracy can be guaranteed for the estimate, if the sample size is large enough.

I'm not sure what this means. General comment: Is it possible to move the figures closer to where they are discussed in the text?

Done. We moved the figures closer to their first mention in the text.

Line 292: Replace "long enough experiments" with "experiments of sufficient length"

Done.

Line 304: "We" should not be capitalized.

Done.

Line 335: Replace “Different to this” with “On the other hand”

Done.

Line 377: Insert: “the” before “one-“

Done.

Line 379: Replace “point” with “points”

Done.

Line 382: Insert “the” before both “Sequential” and “Approximate”

Done.

Line 390: Not sure what “with regarding their weights” means

We rephrased this part of the sentence to clarify that the parameter candidates are selected in consideration of their weights.

Line 405: Insert “a” after “use”

Done.

Line 441: Replace “wrt.” with “with respect to”. This occurs multiple times in the paper. Everyone knows what you mean, but you should probably avoid such an abbreviation in a formal journal article

We replaced every occurrence of ”wrt.” with ”with respect to”, as suggested.

Line 444: Replace “index” by “indices”

Done.

Page 13: I would suggest that you insert a blank line above “Fig 4”. Without this blank line, the “c)” gets lost in the text. It took me a few minutes to figure out which part of your figure was c).

Done.

Line 490: Should “Nested” be capitalized?

We changed it to lowercase.

Lines 504 and 507: Other “wrt” occurrences

Done.

Page 15: See note for Page 13 above.

Done.

Page 16: Table 1 should probably be tabbed to the right. Also , insert “with” before “6” in the first line of the caption

Done. We replaced ”iterations” with the abbreviation ”iter” so that the table fits on the page.

Page 528: Insert “the” before “two”

Done.

Line 556: Another “wrt”

Done.

Line 560: “corollary of that scaling up” should be rephrased

We rephrased that part of the sentence to make clear that the previous mathematical statement directly follows the fact that scaling the rates of a CTMC preserves the transient distribution.

Line 565: "Lemma" shouldn't be capitalized

Done, thank you.

Page 17, caption for Fig 6: Insert "a" before each color

Done.

Page 18: Should Proof 1 be italicized?

Yes, thank you. We changed it.

Line 589: Replace "distribution" with "distributions"

Done.

Line 590: "Lemma" shouldn't be capitalized

Done.

Line 604: The word "lossy" is used. I've never heard of this word.

It is a standard term used in information theory (see e.g. `https://en.wikipedia.org/wiki/Lossy_compression`).

Line 608: Insert "its" before "own"

Done.

Lines 614 – 615: Should "p,q" read "[p,q]"? Should "OK, Failed" read "[OK, Failed]"?

That is true. Thank you.

Line 618: Insert "one" after "allows"

Done.

Caption for Fig 7: Again, insert "a" before all colors

Done.

Line 637: I'm not sure what "over-approximates well" means

We rephrased that sentence to clarify that the SMC-ABC method does capture the viable parameter space, but only as an over-approximation and not the exact values.

Line 648: "ares" should read "areas"

Done.

Line 648: Start a new paragraph with "See"

Done.

Proof 2: Should this be italicized? Replace "equals" with "is equal to"

Done.

Lines 669 – 686: Check the indenting on the second line of each subpoint

Done.

Line 690: Insert "one" after "allows"

Done.

Line 707: Replace “is” with “in”

Done.

Line 714: Replace “to” with “from”

Done.

I hope this helps. Congratulations on a well-written article.

Thank you.

Finally, we would like to comment on the relevant changes we have made in the references. The following original references have been removed:

9 Daca P, et al. Faster statistical model checking for unbounded temporal properties. ACM Transactions on Computational Logic (TOCL). 2017;18(2):12.

10 Ashok P, et al. Statistical Model Checking: Black or White? In: International Symposium on Leveraging Applications of Formal Methods. Springer; 2020. p. 331-349.

The following references have been added:

1 Di Giamberardino P, Iacoviello D. Optimal Resource Allocation to Reduce an Epidemic Spread and Its Complication. Information. 2019;10:213. doi:10.3390/info10060213.

2 Dorigo M, et al. Ant Colony Optimization and Swarm Intelligence. vol. 5217. Springer; 2008.

3 Loreti M, Hillston J. Modelling and analysis of collective adaptive systems with CARMA and its tools. In: Formal Methods for the Design of Computer, Communication and Software Systems. Springer; 2016. p. 83–119.

4 4. Hillston J. Challenges for quantitative analysis of collective adaptive systems. In: International Symposium on Trustworthy Global Computing. Springer; 2013. p. 14–21.

10 Tyson JJ, Csikasz-Nagy A, Novak B. The dynamics of cell cycle regulation. Bioessays. 2002;24(12):1095–1109.

11 Swat M, Kel A, Herzel H. Bifurcation analysis of the regulatory modules of the mammalian G1/S transition. Bioinformatics. 2004;20(10):1506–1511.

16 Raue A, Karlsson J, Saccomani MP, Jirstrand M, Timmer J. Comparison of approaches for parameter identifiability analysis of biological systems. Bioinformatics. 2014;30:1440–1448. doi:10.1093/bioinformatics/btu006.

17 Schnoerr D, Sanguinetti G, Grima R. Approximation and inference methods for stochastic biochemical kinetics—a tutorial review. Journal of Physics A: Mathematical and Theoretical. 2017;50(9):093001.

18 Clarke EM, Henzinger TA, Veith H, Bloem R. Handbook of model checking. vol. 10. Springer; 2018.

19 Bartocci E, Lio P. Computational Modelling, Formal Analysis, and Tools for Systems Biology. PLoS Computational Biology. 2016;12:e1004591. doi:10.1371/journal.pcbi.1004591.
